# Tetrodotoxin‐resistant mechanosensitivity and L‐type calcium channel‐mediated spontaneous calcium activity in enteric neurons

**DOI:** 10.1113/EP091977

**Published:** 2024-07-09

**Authors:** Richard J. Amedzrovi Agbesi, Amira El Merhie, Nick J. Spencer, Tim Hibberd, Nicolas R. Chevalier

**Affiliations:** ^1^ Laboratoire Matière et Systèmes Complexes UMR 7057 Université Paris Cité/CNRS Paris France; ^2^ College of Medicine and Public Health Flinders University Adelaide South Australia Australia

**Keywords:** 2‐APB, adult gut, calcium imaging, Ca_V_1.2, embryonic gut, enteric nervous system, fetal gut, GCamp, L‐type calcium channel, mechanosensation, mouse, tetrodotoxin

## Abstract

**Abstract:**

Gut motility undergoes a switch from myogenic to neurogenic control in late embryonic development. Here, we report on the electrical events that underlie this transition in the enteric nervous system, using the GCaMP6f reporter in neural crest cell derivatives. We found that spontaneous calcium activity is tetrodotoxin (TTX) resistant at stage E11.5, but not at E18.5. Motility at E18.5 was characterized by periodic, alternating high‐ and low‐frequency contractions of the circular smooth muscle; this frequency modulation was inhibited by TTX. Calcium imaging at the neurogenic‐motility stages E18.5–P3 showed that Ca_V_1.2‐positive neurons exhibited spontaneous calcium activity, which was inhibited by nicardipine and 2‐aminoethoxydiphenyl borate (2‐APB). Our protocol locally prevented muscle tone relaxation, arguing for a direct effect of nicardipine on enteric neurons, rather than indirectly by its relaxing effect on muscle. We demonstrated that the ENS was mechanosensitive from early stages on (E14.5) and that this behaviour was TTX and 2‐APB resistant. We extended our results on L‐type channel‐dependent spontaneous activity and TTX‐resistant mechanosensitivity to the adult colon. Our results shed light on the critical transition from myogenic to neurogenic motility in the developing gut, as well as on the intriguing pathways mediating electro‐mechanical sensitivity in the enteric nervous system.

**Highlights:**

- **What is the central question of this study?**
  What are the first neural electric events underlying the transition from myogenic to neurogenic motility in the developing gut, what channels do they depend on, and does the enteric nervous system already exhibit mechanosensitivity?
- **What is the main finding and its importance?**
  ENS calcium activity is sensitive to tetrodotoxin at stage E18.5 but not E11.5. Spontaneous electric activity at fetal and adult stages is crucially dependent on L‐type calcium channels and IP_3_R receptors, and the enteric nervous system exhibits a tetrodotoxin‐resistant mechanosensitive response.

**Abstract figure legend** Tetrodotoxin‐resistant Ca^2+^ rise induced by mechanical stimulation in the E18.5 mouse duodenum.

## 1 INTRODUCTION

The gastrointestinal tract is a tube with the complex task of propelling and mixing bolus to optimize nutrient absorption (Furness, 2007). These functions are made possible by the coordinated action of smooth muscle, interstitial cells and the enteric nervous system (ENS) (Bornstein et al., 2004; Farrugia, 2008; Huizinga et al., 1998; Sanders, 2008). The ENS is an extensive network of neurons, glial and progenitor cells connected to extrinsic autonomic and primary afferent innervation (Sharkey & Mawe, 2023; Spencer & Hu, 2020). The ENS arises from the neural crest cells, mostly from the vagal region of the neural tube (Le Douarin & Teillet, 1973). During embryonic development in mice, neural crest‐derived cells migrate into the developing foregut around embryonic day (E)9.5 and populate it in a wave‐like pattern (Anderson et al., 2006; Kapur et al., 1992). The ENS performs its function by responding to mechanical and chemical cues (Bellono et al., 2017; Bertrand et al., 1997; Kunze et al., 2009).

Myenteric neurons, whether isolated or in situ, can transduce the mechanical forces that act on the gut wall into action potential firing. Such forces include tension, shear‐stress and compression (Mazzuoli & Schemann, 2012; Spencer et al., 2002). K_Ca1_ *_._*_1_ and transient receptor potential (TRP) have been demonstrated to be mechanosensitive channels in enteric neurons (Cavin et al., 2023). Piezo1 and Piezo2 are expressed in enterochromaffin cells of the epithelium and play a major role in shear stress sensing in the lumen (He et al., 2024); Piezo1 is expressed in enteric neurons but it is unclear whether it plays a role in mechanosensation (Ceyhan et al., 2018). Embryonic mouse enteric neurons have been found to respond to electrical field stimulation, and this response was mediated by L‐, N‐, P/Q‐ and R‐type Ca^2+^ channels (Hao et al., 2011). Pharmacological studies have revealed that the main intestinal motility patterns in mature animals – segmentation, peristalsis and migrating motor complex – all depend on neuronal activity (Gwynne et al., 2004; Hasler, 2009). In the mouse embryo, the first motor activity at E13.5 is purely myogenic and takes the form of spontaneous circular muscle contractile waves that propagate along the intestine (Chevalier, Agbesi et al., 2021; Roberts et al., 2010). The myenteric plexus is present throughout the gut from E14.5 but does not influence contractility until E18.5, when the first neurogenic motility patterns can be detected (Roberts et al., 2007, 2010). The developmental transition from myogenic to neurogenic patterns yields great insight into the basic mechanisms by which the ENS affects motility.

In the chicken embryo, our group has found that the ENS gives rise to a descending, inhibitory, mechanosensitive coupling of the circular and longitudinal muscle layers (Chevalier et al., 2019). This antagonism gives rise to a new motor pattern, the migrating motor complex (MMC). The MMC has the same frequency as longitudinal smooth muscle contractions, which act as a trigger for this motor pattern. The polar, descending character of neural inhibition gives rise to the stereotyped ‘ascending contraction–descending inhibition’ response to mechanical stimulation, known as ‘the law of the intestine’ (Bayliss & Starling, 1899). We wondered to what extent the results we obtained in chicken applied to a mammalian model, the mouse. We took advantage here of a mouse model expressing the GCaMP6f fluorescent intracellular Ca^2+^ reporter in neural crest‐derived cells. We studied the development of ENS Ca^2+^ activity from E11.5 through postnatal day (P)3; late fetal and early postnatal Ca^2+^ imaging of the ENS has to the best of our knowledge not been examined previously. We were in particular keen on preserving an intact muscular activity (imaging without muscle inhibitors like nicardipine) to observe the coupling of contractions with neuronal activity. The embryonic gut is small, enabling the study of large ENS areas and network‐wide effects that may not be evident from visualizing only a few ganglia, as is typical of adult gut studies. Because ENS electrical activity in the embryo was sensitive to mechanical stimulation, we investigated which ion channels could relay this mechanosensitive response, and extended some our more salient results to the adult colon.

## 2 METHODS

### 2.1 Ethical approval

All experiments were carried out according to the guidelines of CNRS and INSERM animal welfare committees, and conform to their principles and regulations. Mice were hosted at the Institut Jacques Monod animal husbandry, and had access to housing and food and water ad libitum. Temperature in the husbandry was 20–24°C and humidity in the range 45–55%. The mice were exposed to light from 07.00 to 19.00 h. Females were kept in common cages with a maximum of five females per cage. For matings, the male was introduced in the evening and removed in the morning after detection of plugs in the morning. Pregnant mice were killed by cervical dislocation to retrieve embryos age E11.5–E18.5. The embryos were separated and immediately beheaded with surgical scissors. Pups at P1 and P3 were beheaded with surgical scissors. The methods used to kill the mice/pups/embryos conform to the guidelines of CNRS and INSERM animal welfare committees. Killing of mice for retrieval of embryos and/or their gastrointestinal tract is a terminal procedure for which neither CNRS nor INSERM assign ethics approval codes and hence none are given here.

### 2.2 Animal model

The Cre‐dependent B6J.Cg‐Gt(ROSA)26Sortm95.1(CAG‐GCaMP6f)Hze/MwarJ transgenic mouse line (referred to as Ai95(RCL‐GCaMP6f)‐D (C57BL/6J)) expresses a floxed‐STOP cassette blocking transcription of the fast Ca^2+^ indicator GCaMP6f and has been characterized previously (Madisen et al., 2010). We refer to them as GCaMP fl/fl. A transgenic mouse line in which the transgene is under the control of the 3‐kb fragment of the human tissue plasminogen activator (Ht‐PA) promoter Tg(PLATcre) 116Sdu16 is referred to as Ht‐PA::Cre (Chevalier, Ammouche et al., 2021; Pietri et al., 2003). GCaMP fl/fl males were crossed with Ht‐PA::Cre females to generate embryos carrying a Ca^2+^ fluorescent reporter in neural crest cells and their derivatives (neurons, glia, progenitors). The day a plug was observed following mating overnight was defined as E0.5. The day of birth was considered as P0 = E19.5. All mice were born at E19.5. The mice were dissected at E11.5, E14.5, E18.5, P1 and P3. The gastrointestinal tracts were dissected from the stomach to the hindgut in phosphate‐buffered saline (PBS) with Ca^2+^ and Mg^2+^, and the mesentery was removed. While most embryos expressed GCaMP only in the ENS, we observed that in approximately ∼20% of the embryos GCaMP could also be expressed in smooth muscle cells, as can be observed in, for example, Supporting information Video S14. In this case the signals emanating from the ENS or from smooth muscle could readily be distinguished on a morphological basis, but we did not use those samples for quantitative transient analysis. Some samples exhibited expression solely in the smooth muscle and not in the ENS and were not included in this study.

### 2.3 Calcium imaging and analysis

After dissection, a 0.2 mm‐diameter nylon thread was inserted in the lumen of an ∼2 cm long gut segment. The end of the thread was inserted in a block of soft PDMS (Sylgard 184) in a way that the nylon gently pressed the gut segment against the bottom of a 35 mm‐diameter Petri dish. This method has been detailed in (Chevalier et al., 2020). It prohibits bulk movements of the sample in and out of the imaging field due to smooth muscle contractions. The gut was covered with 5 mL of Dulbecco's modified Eagle's medium(DMEM) supplemented with 1% penicillin and constantly bubbled with 95% O_2_–5% CO_2_ at 36.5 ± 1°C. The gut was imaged with an inverted spinning disk confocal microscope (Olympus or Zeiss, France, W1 at the Imagoseine platform) with ×10 objective, and illumination wavelength was set to 488 nm. Fluorescence was collected in the range 512–630 nm, and captured with an iXon 512 × 512 px camera (Andor, Ireland) or a Zyla 1040 × 1392 px camera (Andor, Ireland) at a rate of 2 Hz. Video duration was 2–3 min. Drugs were administered from a stock solution by directly adding them to the Petri dish under the microscope. Drugs used and stock concentrations were: GdCl_3_ (Tocris, UK, 4741, 50 mM in water), nicardipine (Sigma, France, N7510, 10 mM in dimethyl sulfoxide (DMSO)), RyR (Tocris, UK, 1329, 10 mM in DMSO), tetrodotoxin (TTX; Abcam, Cambridge, UK, 120055, 1 mM in water), NOLA (SCBT, Germany, 222066, 5 mM in water), veratridine (Sigma, France, V5754, 20 mM in DMSO), 2‐aminoethoxydiphenyl borate (2‐APB; Tocris 1224, 100 mM in DMSO). To ensure proper mixing, the solutions were gently agitated by up‐and‐down movements using a 1 mL pipette. Synaptic block solution was prepared by adding 0.25 mM CaCl_2_ and 10 mM MgCl_2_ to DMEM without calcium and magnesium. For the smaller E11.5 guts that do not necessitate extra‐oxygenation, a different procedure was adopted: they were immobilized in 1 mL 1% low‐melting point agarose (Sigma, France, A4018) after dissection, covered with 2 mL medium and imaged in 95% air–5% CO_2_ at 36.5 ± 1°C.

To analyse Ca^2+^ transients, a region of interest (ROI) was selected over the entire ENS area that was in the confocal plane. This ROI was divided into cell‐sized square‐boxes using the ImageJ Grid‐ROI macro. The pixel intensity change over time in each box was measured with the ‘Multi Measure’ function. Peaks in the intensity versus time data (i.e., Ca^2+^ transients) of each square box were obtained using the ‘findpeaks’ function in MATLAB (MathWorks, Natick, MA, USA), setting optimum and constant: minimal peak prominence, minimal and maximal peak width. These thresholds allowed us to filter noise, and changes due to sample drift or slight movements. One spontaneous event giving rise to one peak in an analysis box was considered as one single calcium transient. We next assessed the area of ENS (mm^2^) in the imaging field using the intermodes thresholding method in Fiji. Normalized Ca^2+^ activity was finally reported as the total number of peaks per unit area ENS per unit time, that is, in Hz/mm^2^. In one experiment (Figure 2e) where we did not press the gut against the Petri dish bottom, contractile movements of the gut caused movement of the ganglia in‐and‐out of the ROI and we thus couldn't apply the MATLAB routine described above. We therefore counted Ca^2+^ transients in this experiment by eye, focusing on one ganglion at a time, summing up all the counts from the different ganglia in the field of view. The total number of events in the recordings was normalized by the ENS area as described above.

For lumen‐pressurization experiments (Figure 4m, Video S11), a blunted G25 needle was inserted in a hole at the base of a 35 mm Petri dish and the hole–needle junction sealed with silicone paste (CAF4). Guts were then cannulated on the blunted G25 liquid‐filled needle, tying two tight knots with elastane thread, one at the free end (caudal), one at the needle–gut interface (rostral). The gut was not pressed against the Petri dish bottom so that lumen inflation could freely expand the lower gut wall. The needle was then connected to a 1 mL syringe containing medium and placed in the 37°C atmosphere of the confocal microscope (Olympus). A small amount of liquid was injected in the lumen of the gut while performing Ca^2+^ imaging and the applied distension was quantified by computing the elongation of ganglia before/after pressurization.

### 2.4 Immunohistochemistry

Samples were fixed for 1 h in 4% paraformaldehyde in PBS, and washed 3 times. They were then dehydrated for 24 h in 30% sucrose solution in water. Segments of length 1–2 cm were embedded in OCT and frozen with a −80°C freezer pack. Frozen longitudinal sections were performed on a Leica, Switzerland cryostat set to −20°C, at 14 μm thickness. Thin slice sections were blocked and permeated in 1% BSA and 0.1% Triton X‐100 in PBS overnight. The following primary antibodies were applied overnight: anti‐ Ca_V_1.2 (1:100, ACC003AN7125, Alomone Labs, Jerusalem, Israel), Calretinin (1:200, 6B3, Swant, Switzerland). After washing, the following conjugated antibodies were applied overnight: Choactase (1:100, sc‐55557 AF 546 conjugated, SCBT) and nitric oxide synthase 1 (NOS1; 1:100, sc‐5302 AF 647 conjugated, SCBT). They were combined with the necessary secondary antibodies (1:400) for the non‐conjugated Ca_V_1.2 and Calretinin antibodies.

### 2.5 Statistics and reproducibility

**FIGURE 1:**
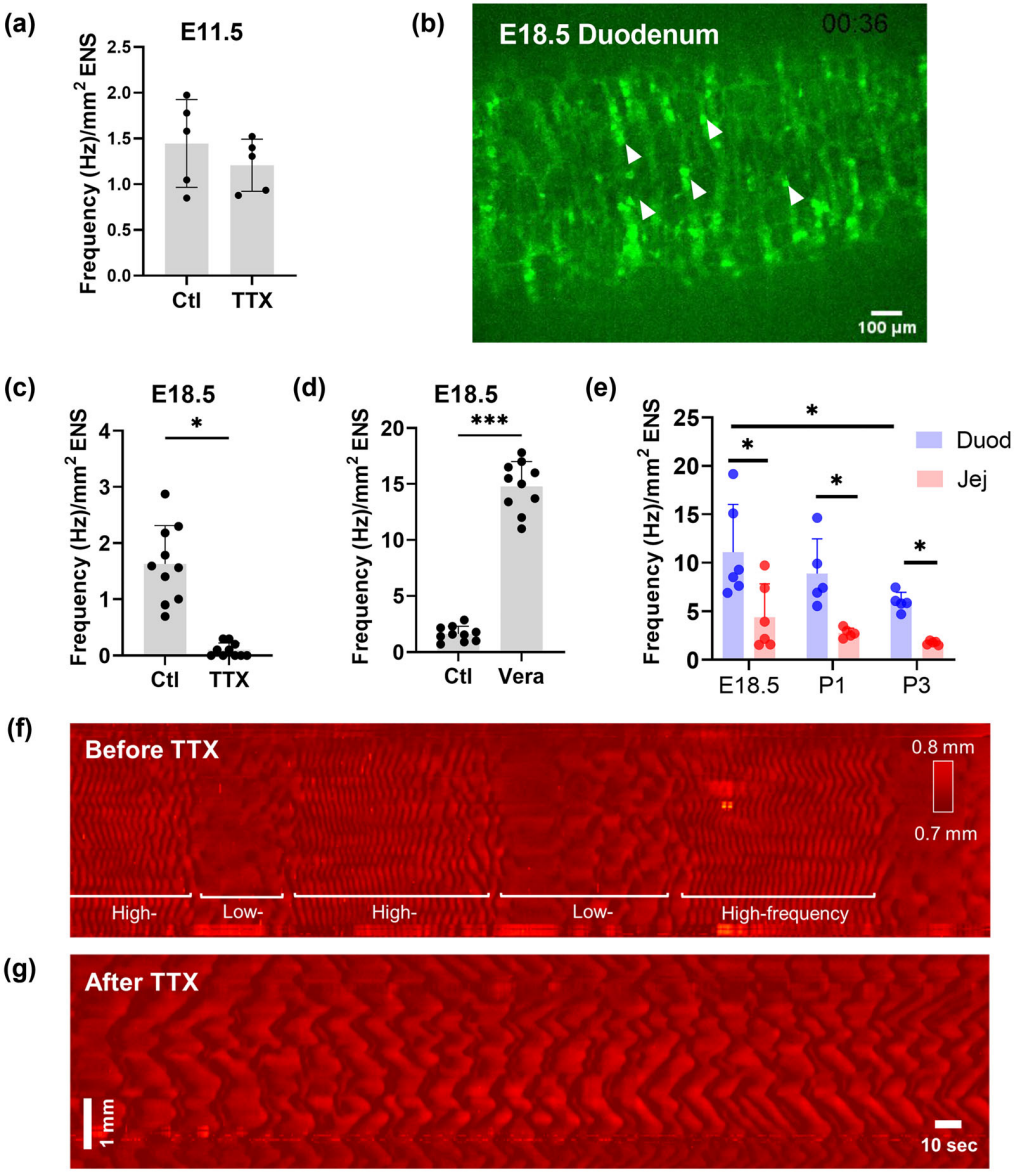
Tetrodotoxin sensitivity of Ca^2+^ activity and motility. (a) Spontaneous ENS Ca^2+^ activity in E11.5 ileum and after addition of TTX (1 μM). (b) Spontaneous ENS Ca^2+^ activity in E18.5 duodenum; white arrowheads point at select Ca^2+^ transients. (c,d) Spontaneous ENS Ca^2+^ activity in E18.5 duodenum, before and after addition of TTX (1 μM, *P* = 0.0427, *t*‐test) or veratridine (10 μM, *P* = 0.00008, *t*‐test). (e) Evolution of Ca^2+^ activity from E18.5 to P3 in the duodenum and jejunum (*P* = 0.011, 2‐way ANOVA). (f) Spatio‐temporal diameter map showing an alternating high‐ and low‐frequency circular smooth‐muscle contraction pattern in E18.5 duodenum, detected in *n *= 4/10 samples. (g) This pattern is abolished after TTX (1 μM) application.

**FIGURE 2:**
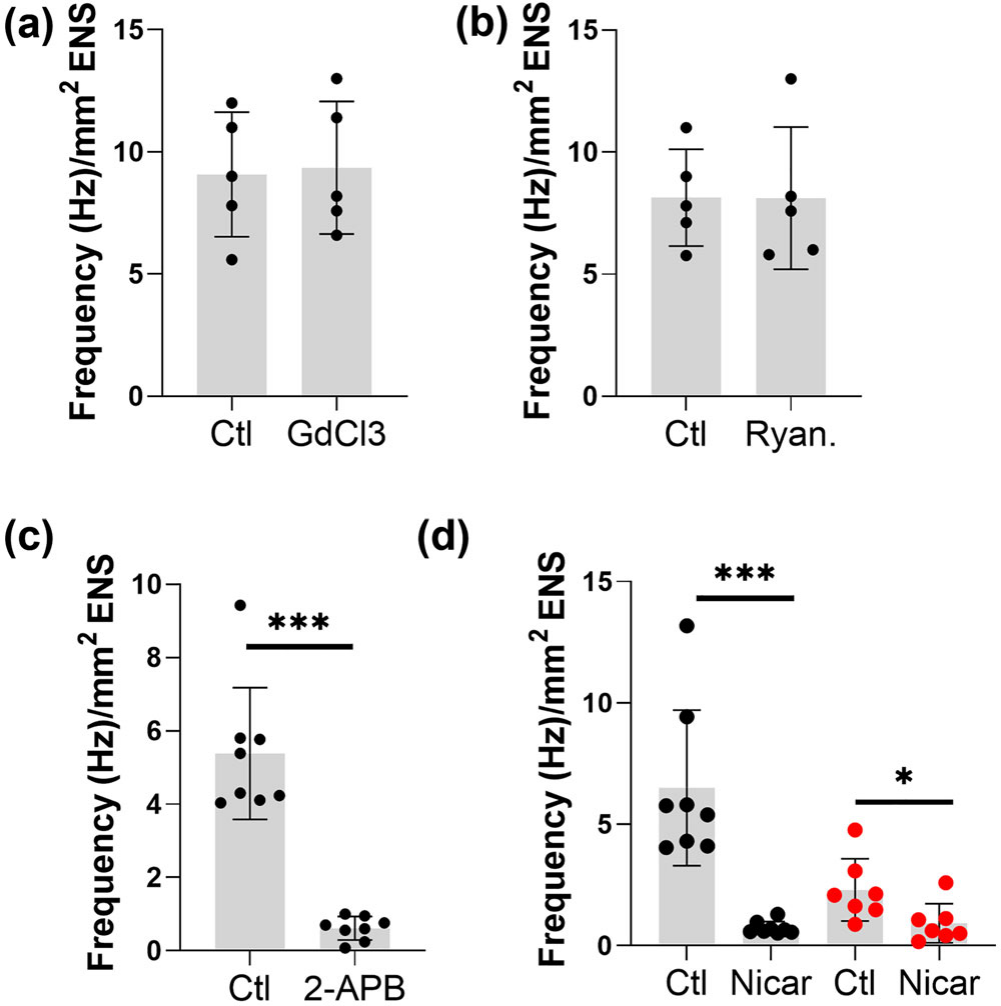
Pharmacological perturbation of Ca^2+^ activity in E18.5 duodenum. Effects of (a) GdCl_3_ (100 μM, *n *= 5), (b) ryanodine (10 μM, *n *= 5), (c) 2‐APB (20 μM, *n *= 8); *P *< 0.0001, *t*‐test, and (d) nicardipine (10 μM) when the gut segment was pressed against the Petri dish bottom (black dots, *n *= 8) and when not (i.e. free to contract, *n *= 7, red dots). ***P = 0.00017, *P = 0.032, *t*‐test.

All sample numbers indicated in this article correspond to different embryos (guts, biological replicates). For mice, data were collected from a minimum of three different litters per age group to ensure robustness. Each group presented in this study comprises a minimum of *n* = 5 samples, ensuring an adequate sample size for statistical analysis. Statistical analysis was performed with 2‐way ANOVA and Student's *t*‐test, and exact sample numbers and *P*‐values are reported in the figure legends. Bars in Figures 1, 2 and 5 indicate the mean and standard deviation.

## 3 RESULTS

### 3.1 Tetrodotoxin sensitivity of Ca^2+^ activity and motility at embryonic and fetal stages

Ca^2+^ activity in differentiating neurons and enteric neural crest cells has been reported at early stages (E11.5–E14.5), but was found to be absent at E16.5 (Hao et al., 2017). These authors found that purinergic receptors were involved in the generation of Ca^2+^ transients, suggesting that this early Ca^2+^ activity might fundamentally depend on different channels than mature, Na^+^ channel‐dependent activity. We tested this and found that tetrodotoxin (TTX, 1 μM) did not affect Ca^2+^ activity at E11.5 (Figure 1a, Video S1). Because the first neurogenic motility patterns have been reported to take place at E18.5 (Roberts et al., 2010), we next characterized Ca^2+^ activity at this stage. Ca^2+^ signals at E18.5 were detected in discrete nerve cell bodies within single ganglia, but their activation was not temporally synchronized across ganglia, nor as a propagating wave (Figure 1b, Video S2). Ca^2+^ activity was abolished by TTX (Figure 1c, Video S2) and was conversely increased more than 10‐fold by veratridine (Figure 1d, Video S2), a depolarizer that causes persistent opening of the voltage‐gated Na^+^ channels. These results indicate that Ca^2+^ signals at E18.5 stem from mature, Na^+^ channel‐dependent enteric neural electric activity. We next examined how Ca^2+^ activity evolved from E18.5 to P3 in the duodenum and jejunum. Ca^2+^ activity decreased from E18.5 to P3 in both the duodenum and jejunum (Figure 1e); this decrease was statistically significant for the duodenum between E18.5 and P3. The Ca^2+^ activity in the duodenum was always a factor of ∼2 higher than in the jejunum at each stage considered (Figure 1e).

We next determined how TTX affected motility when the first spontaneous neurogenic activity sets in at E18.5. Motility at earlier stages (E13.5–E16.5) has been previously shown to be purely myogenic (Roberts et al., 2010). Motility at E18.5 was characterized by propagating circular contractions and pendular longitudinal contractions. The duodenum (*n* = 4/10), and jejunum (*n* = 5/10) showed alternating high (>18 cpm) and low (<10 cpm) frequency circular contractions (Figure 1f, Video S3). The periods of high‐frequency contractions occurred at a rate of 0.55 ± 0.1 cpm and lasted for 60 ± 5 s. After TTX was added, the alternating high‐ and low‐frequency wave pattern was abolished (Figure 1g, Video S3). The gut in TTX displayed rhythmic contractions with a frequency of 11 ± 2 cpm, that is, between the high and low dominant frequencies before TTX was introduced.

### 3.2 Fetal spontaneous Ca^2+^ activity is IP_3_R‐ and Ca_V_1.2 channel‐dependent

We next investigated the molecular mechanisms involved in spontaneous Ca^2+^ spike generation at E18.5. GdCl_3_ (100 μM, *n* = 5), a transient receptor potential (TRP) channel blocker, and ryanodine (10 μM, *n* = 5), a blocker of the RyRs at the endoplasmic reticulum, had no effect on Ca^2+^ activity (Figure 2a,b). 2‐APB (20 μM, *n* = 8), a known inhibitor of inositol 1,4,5‐trisphosphate receptor (IP_3_R) and store‐operated Ca^2+^ channels (SOC) significantly reduced spontaneous Ca^2+^ activity (Figure 2c, Video S4). Surprisingly, nicardipine (10 μM, *n *= 8), an L‐type Ca^2+^ channel blocker, almost completely abolished spontaneous Ca^2+^ activity (Figure 2d black circles, Video S5). This effect could be mediated by a direct effect of nicardipine on enteric neurons, or by an indirect effect of tone release in the smooth muscle, driving a decrease of Ca^2+^ activity in the myenteric plexus. We did not, however, observe any distension or morphological changes of the ENS caused by nicardipine‐induced tone release, as the bottom of the sample was pressed against the observation window: this suggests that our protocol inhibited tone release locally, by preventing in‐plane movement of the circular and longitudinal muscle, in the region we imaged. We confirmed this absence of morphological changes after nicardipine application in all samples examined (*n *= 8), a representative example of which can be seen in Video S5. In contrast, when samples were not pressed against the Petri dish bottom, nicardipine clearly abolished muscle contractions (*n* = 7/7). The mean change of Ca^2+^ activity before/after nicardipine for each sample was −60 ± 15% (*n *= 7, Figure 2d red circles, Video S6). This decrease was significant, but less important than in the ‘pressed’ state.

**FIGURE 3:**
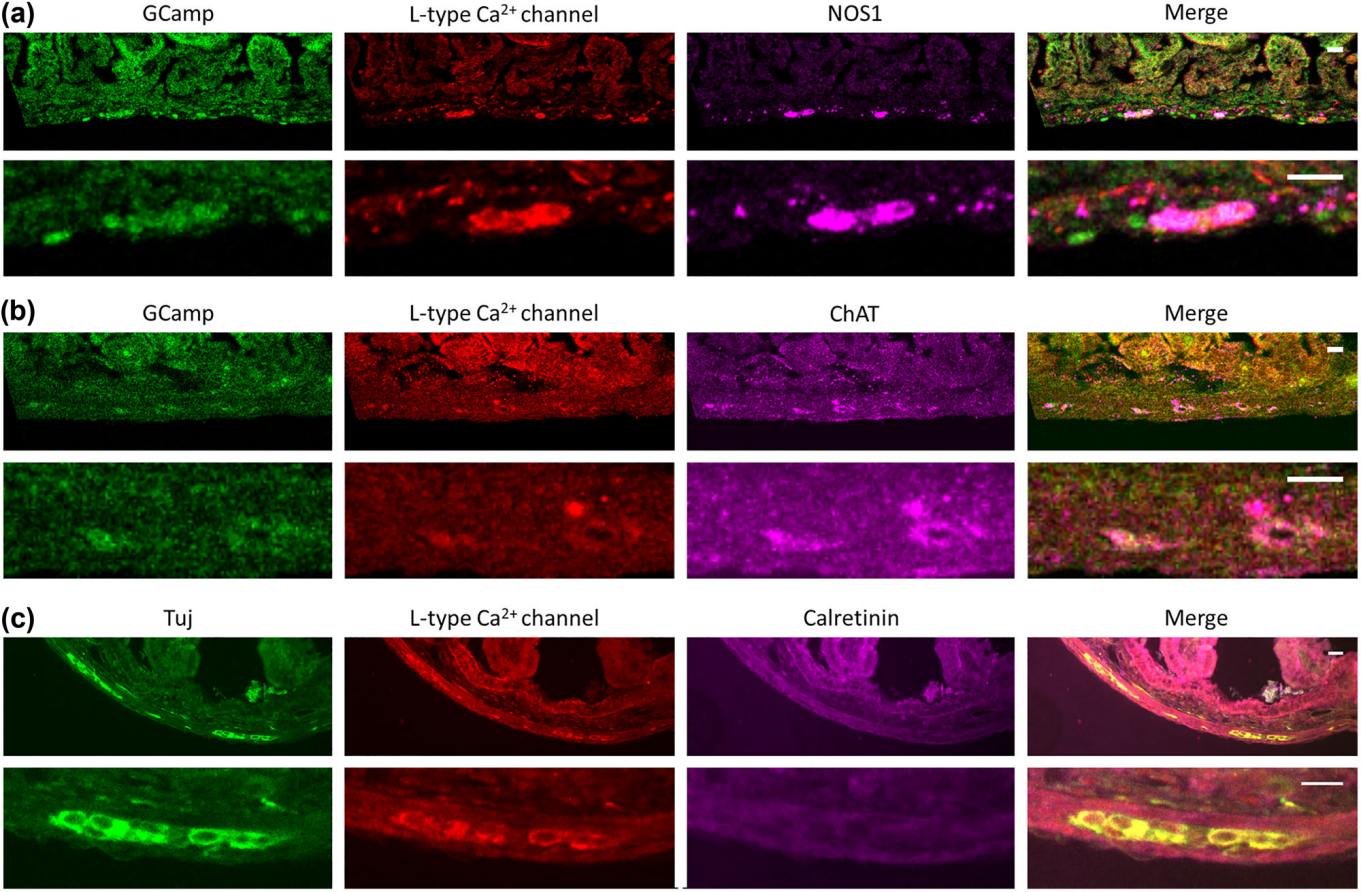
Presence of L‐type Ca^2+^ channels on enteric neurons in E18.5 duodenum. (a) GCaMP‐NOS1, (b) GCaMP‐ChAT, (c) Tuj‐Calretinin. For each series, two rows (low‐magnification and high‐magnification) are presented; the scale bar is 20 μm in all images. NOS1 staining was more conspicuous: the linear density along the length of the gut periphery of NOS1 and ChAT was respectively 44 ± 10%, and 7 ± 3% (*n* = 9 slices). Calretinin staining was absent in all slices (*n* = 8 slices) examined. Similar results were obtained in *n* = 4 embryos.

The possibility that nicardipine could directly act on neurons was further corroborated by immunohistochemistry (IHC) for L‐type Ca^2+^ channels. IHC revealed conspicuous L‐type voltage‐gated Ca^2+^ channels (Ca_V_1.2) on myenteric GCaMP (Figure 3a,b) or βIII‐tubulin positive cells (Figure 3c); Ca_V_1.2 staining appeared in fact more intense than on neighbouring smooth muscle cells (Figure 3a–c). We co‐labelled with nitric oxide synthase (NOS) for inhibitory neurons and choline acetyltransferase (ChAT) and calretinin for excitatory neurons. Calretinin has been shown to be highly expressed in ChAT^+^ neurons in small intestine (Qu et al., 2008) and colon (Feng et al., 2022). Whereas many neurons appeared NOS1‐positive (Figure 3a), we could only detect a small population of ChAT‐positive neurons at E18.5 (Figure 3b) and no calretinin immunoreactive neurons (Figure 3c). Both ChAT‐ and NOS1‐positive neurons co‐labelled for Ca_V_1.2 (Figure 3a,b). Although our IHC results suggest a higher degree of inhibitory neurons than cholinergic ones in E18.5 duodenum, a previous study using a ChAT reporter mouse has found that cholinergic and NOS neurons are present in approximately equal amounts (∼20% each) at P0 (Hao et al., 2013).

### 3.3 E14.5 and E18.5 ENS exhibits TTX‐ and 2‐APB‐resistant mechanosensitivity

**FIGURE 4:**
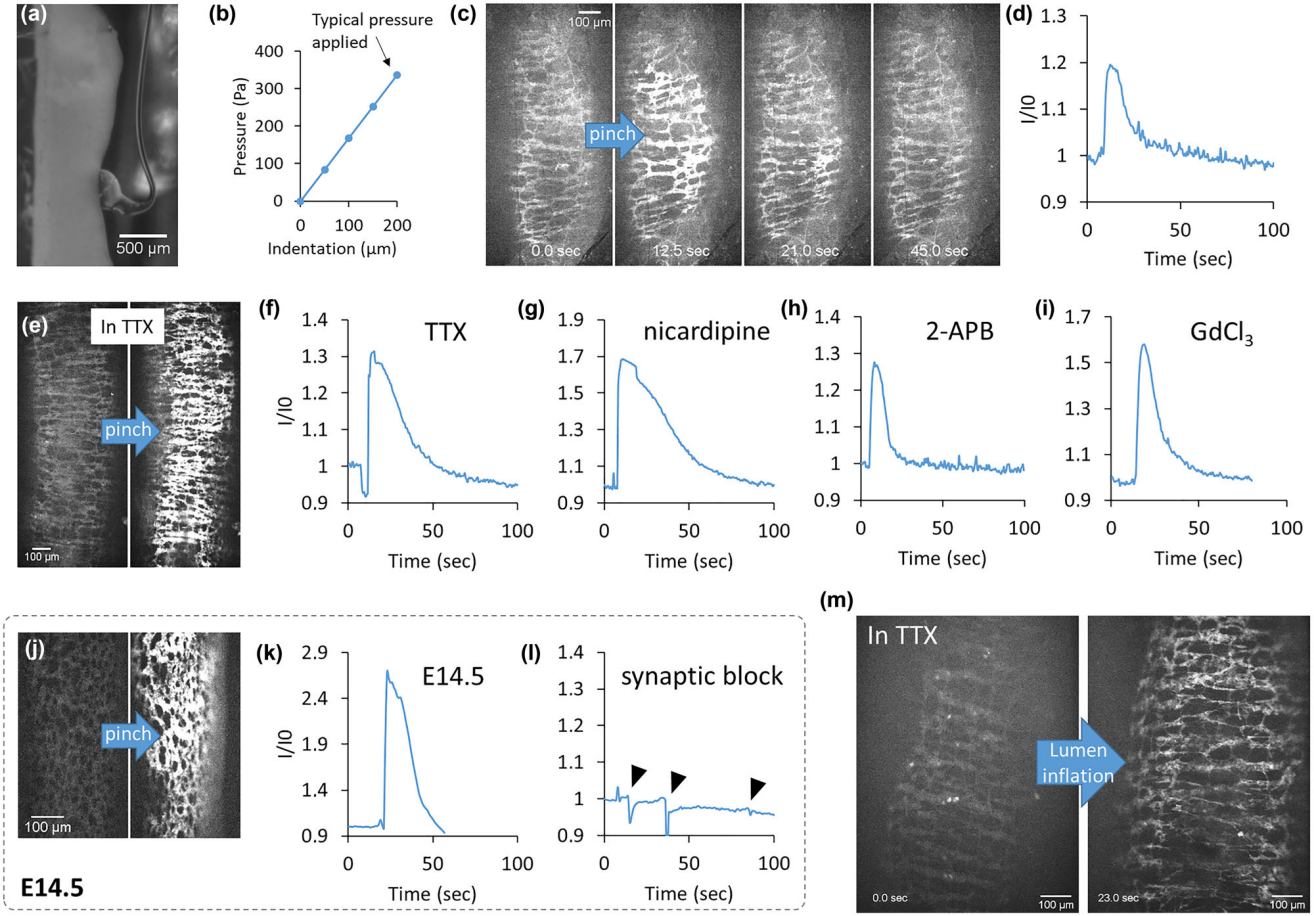
Ca^2+^ up‐rise induced by mechanical compression or lumen inflation. All data are from E18.5 duodenum except (j–l) which was an E14.5 jejunum. (a) Lateral view of the ‘pinched’ gut indented by a ∼300 μm glass sphere (Video S7). (b) Indenter pressure as a function of indentation depth. The gut was typically indented ∼200 μm in subsequent tests, that is, at pressures around ∼400 Pa. (c) Select frames from Video S8 showing Ca^2+^ rise induced by pinching in control conditions (*n *= 10) and (d) time trace of relative fluorescence intensity in the dashed yellow ROI during pinching. (e,f) Pinch‐induced Ca^2+^ rise persists in the presence of TTX 1 μM (*n *= 8), (g) nicardipine 10 μM (*n *= 8), (h) 2‐APB 100 μM (*n *= 8) and (i) GdCl_3_ 100 μM (*n *= 5, Video S10). (j,k) Pinch‐induced Ca^2+^ rise could be elicited at stage E14.5 (*n *= 3), but (l) was inhibited in synaptic block solution (*n *= 3). (m) Lumen inflation also elicited a transient Ca^2+^ rise in the presence of TTX 1 μM (*n *= 5, Video S11); distension of the ENS ganglia by pressure can be clearly seen.

The ENS is known to be mechanosensitive (Cavin et al., 2023; Huizinga et al., 1998; Kunze et al., 1998) and we were therefore curious to find out whether such properties are already established at embryonic or fetal stages. To this end, we devised a mechanical force compression assay, consisting of a 0.3 mm‐diameter blunt glass probe to gently pinch the gut wall (Figure 4a, Video S7). We measured the pressure applied to the gut wall from the deflection of a force‐calibrated pipette to be ∼400 Pa at 200 μm indentation (Figure 4b), consistent with typical elastic moduli of ∼2 kPa of the gut at embryonic stages (Chevalier et al., 2016). Subsequent pinches were performed at similar indentation values, but manually under the confocal microscope; the value of 400 Pa therefore only represents an estimate of the pressure applied. Intracellular Ca^2+^ concentration in the ENS promptly rose to high levels upon pinching (Figure 4c,d, *n* = 10/10, Video S8). We also observed contraction of the circular smooth muscle (*n* = 7/10 at E18.5) that was synchronous with the intracellular Ca^2+^ rise in the ENS (Video S8). This effect could be reproduced repeatedly (three subsequent pinches within 3 min, Video S9) in the same ENS area, indicating that it did not result from ENS wounding induced by the pinch.

We next investigated which ion channels and molecular pathways could mediate the mechanosensitive behaviour of the E18.5 ENS. The mechanosensitive Ca^2+^ up‐rise persisted in the presence of the three compounds that abrogated spontaneous Ca^2+^ activity, TTX (1 μM, *n *= 8, Figure 4e,f, Video S10), nicardipine (10 μM, *n *= 8, Figure 4g, Video S10) and 2‐APB (100 μM, *n *= 8, Figure 4h, Video S10). We further tested NOLA (100 μM, *n *= 5), an inhibitor of nitric oxide synthase, and GdCl_3_(100 μM, *n *= 5, Figure 4i, Video S10); neither of these compounds inhibited the mechanosensitive Ca^2+^ up‐rise. We found that the same mechanosensitive response was also present at an earlier developmental stage, at E14.5 (Figure 4j and k). The only condition that abolished the pinch‐induced Ca^2+^ response was when samples (E14.5, *n *= 3, Figure 4l) were incubated in a synaptic block solution at Mg^2+^ 10 μM and Ca^2+^ 0.25 μM (Dean & Boulant, 1989); this solution also abolished spontaneous ENS Ca^2+^ activity. We further found a Ca^2+^ up‐rise could be elicited in TTX by lumen pressurization (*n *= 5, Figure 4m, Video S11, Methods) a more physiological mechanical stimulation method. Ganglia distension after pressurization was 41 ± 16% (*n *= 5).

We note that our mechanical stimulation assays did not allow for quantitative comparison of the intensity of the Ca^2+^ up‐rise between different guts/pharmacological conditions, because it often induced slight sample displacement out of the confocal plane, necessitating on‐the‐spot readjustment. In addition, pinching was performed manually, and therefore did not allow for a precise control of strain around the ∼400 Pa value, or of strain rate.

### 3.4 Adult colon spontaneous Ca^2+^ activity is Ca_V_1.2 channel‐dependent and displays TTX‐resistant mechanosensitivity

**FIGURE 5:**
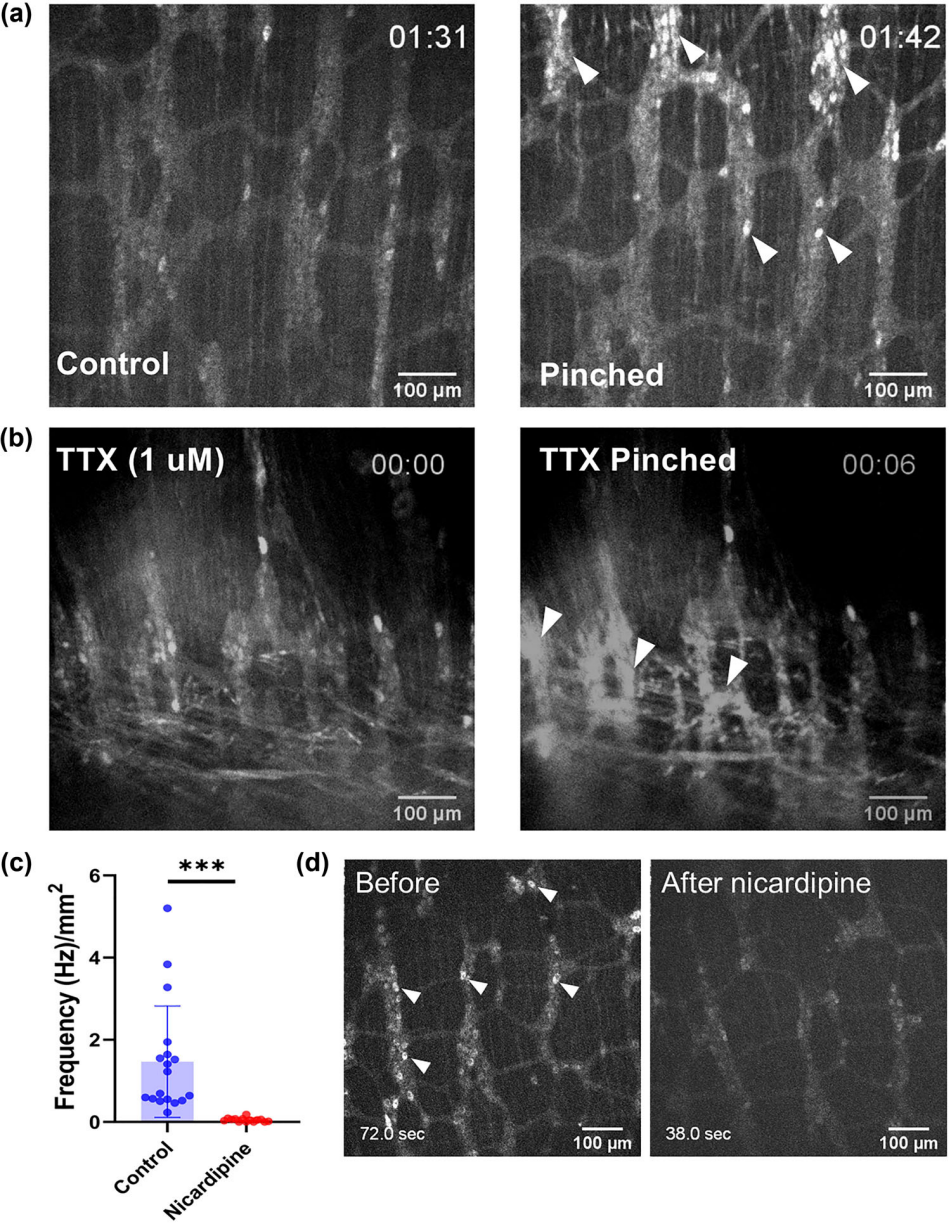
TTX‐resistant mechanosensitivity and nicardipine sensitivity in the adult mouse colon. (a) Ca^2+^ activity of adult mouse colon before and when pinched (Video S12). (b) Ca^2+^ activity in the presence of TTX (1 μM) before and when pinched (Video S13). (c,d) Effects of nicardipine (10 μM) on spontaneous calcium activity (*n *= 7 different animals, *n *= 18 different segments; Video S15; *P* = 0.00008, *t*‐test). Arrowheads point to select Ca^2+^ transients.

We finally investigated whether TTX‐resistant mechanosensitivity and the inhibiting effects of nicardipine on Ca^2+^ activity were specific to embryonic stages, or could also be present in adult gut. The duodenum presented contractions too strong for it to be held in place by the nylon‐thread‐in‐lumen method (Methods), making visualization of Ca^2+^ changes impractical. We therefore applied our pinching protocol to the less motile adult colon. We observed an instantaneous increase in Ca^2+^ activity (Figure 5a, Video S12) in the ENS (*n* = 3) upon pinching, which persisted after addition of TTX (1 μM) (*n *= 3, Figure 5b, Video S13; note that the region of the gut pinched is a few millimetres away from the point imaged). Blocking synaptic transmission (Mg^2+^ 10 μM, Ca^2+^ 0.25 μM), as we found at E14.5, blocked the pinch‐induced Ca^2+^ rise; it also significantly reduced spontaneous Ca^2+^ activity (Video S14, *n *= 3). Contractions of the smooth muscles, however, persisted when synaptic transmission was blocked and a pinch‐induced contraction of the smooth muscles could be elicited (*n* = 1/3).

We finally found that nicardipine (10 μM), as in the fetal mouse, almost completely abolished spontaneous ENS Ca^2+^ activity in the adult colon (Figure 5c,d, n = 7, Video S15).

## 4 DISCUSSION

In this study, we showed that TTX does not affect spontaneous Ca^2+^ activity at E11.5, but abolishes it at the neurogenic motility stage E18.5 (Figure 1a–c). The frequency of this activity decreased in the fetal and early postnatal (E18.5–P3) mouse ENS (Figure 1e). We detected a neurogenic motility pattern of alternating high‐ and low‐frequency circular smooth muscle contractions at E18.5 (Figure 1f,g). Spontaneous Ca^2+^ activity required intracellular IP_3_R store‐operated Ca^2+^ channels (Figure 2c, Video S5), and was almost completely abolished or diminished by nicardipine at E18.5 (Figure 2d, Video S6). Nicardipine also inhibited spontaneous Ca^2+^ activity in adult colon (Figure 5c,d, Video S15). L‐type Ca^2+^ channel immuno‐reactivity was identified in both NOS1 and ChAT enteric neurons (Figure 3). Muscle tone‐related changes in ENS morphology were minimal when samples were pressed against the observation window, suggesting together with our IHC results that nicardipine could directly act on ENS neurons. We finally discovered that the fetal ENS can transduce mechanical force changes (pinching, lumen pressurization) into a fast, massive, transient Ca^2+^ up‐rise (Figure 4). This behaviour was present as from E14.5 (Figure 4j,k), was TTX‐resistant both in the fetal (Figure 4e,f) and the adult gut (Figure 5a,b), but was inhibited in low‐calcium high‐magnesium conditions (Figure 4l, Video S14).

Spontaneous Ca^2+^ activity has been recorded in many parts of the developing central nervous system and is known to control essential aspects of development (Ben‐Ari et al., 1989; Gu & Spitzer, 1995; Gust et al., 2003; Holliday & Spitzer, 1990; Milner & Landmesser, 1999; Wong et al., 1995). We confirmed the presence of spontaneous Ca^2+^ activity at colonization stage E11.5 (Hao et al., 2017). Interestingly, we found that Ca^2+^ activity was insensitive to TTX in the ileum at E11.5. This may indicate that early Ca^2+^ activity stems mainly from non‐differentiated enteric neural crest cells, which relies on distinct channels from mature neuronal activity. Spontaneous ENS Ca^2+^ activity was found not to be correlated with gut contractions in the period E13.5–E14.5 and then vanished at stage E16.5 (Hao et al., 2017). Neurites are, however, already capable of transmitting electrical information upon luminal chemical stimulation at E15.5 (Hao et al., 2020). The first neurogenic motility patterns emerge at E18.5 (Roberts et al., 2010). We detected TTX‐dependent Ca^2+^ activity in E18.5 duodenum and jejunum, corroborating and complementing previous motility (Roberts et al., 2010) and electrophysiological studies (Foong et al., 2012; Hao et al., 2012). We found that the first neurogenic motility pattern was characterized by alternating high‐ and low‐frequency circular smooth muscle contractile waves in the duodenum and jejunum. This pattern could correspond to the ‘contraction complexes’ found in Roberts et al. (2010), although this is not completely clear from comparing the space–time diagrams and our respective descriptions. As this neurally mediated motility pattern develops just prior to birth, it could facilitate absorption of milk‐derived nutrients during weaning; it might prefigure neurogenic motility patterns like segmentation or the migrating motor complex that are crucial in mature animals (Bornstein et al., 2004; Furness, 2007).

We found that Ca^2+^ activity was higher in the duodenum than in the jejunum at each of the stages considered (E18.5, P1, P3). Because enteric neurons colonize the duodenum first and mature earlier in more rostral regions of the gut (Kapur et al., 1992; Young et al., 1998), this higher activity in the duodenum could result from increased ion channel expression or developmental changes in ion channel properties: neurogenic motility in the hindgut for example only develops from stage P10 (Roberts et al., 2007); the amplitude of Ca^2+^ response to electrical stimulation from E15.5 and E18.5 duodenum has been reported to be higher than those from the colon at the same ages (Hao et al., 2011). Unexpectedly, we found that spontaneous Ca^2+^ activity decreased from E18.5–P3 in the duodenum and jejunum. This observation parallels studies in the central nervous system showing that spontaneous depolarizing potentials are observed during the first eight postnatal days in the hypothalamus, but decrease from P9 to P12 and disappear by P12.2 (Ben‐Ari et al., 1989).

L‐ type Ca^2+^ channels are prominently expressed in smooth muscle (Bodi et al., 2005; Lacampagne et al., 1994), and dihydropyridine inhibitors are often used in electrophysiological and Ca^2+^ imaging studies of the ENS to limit movements of intestinal preparations. Our protocol constrained movements by gently pressing the gut against the Petri dish bottom, and we were thus able to measure the effects of nicardipine on spontaneous neural activity. Strikingly, this compound inhibited spontaneous activity, both in the fetal E18.5 mouse duodenum and in the the adult mouse colon. We found that L‐type Ca^2+^ channels are expressed by enteric neurons, even more conspicuously than on smooth‐muscle. These findings are consistent with previous investigations reporting L‐type Ca^2+^ channels on both embryonic (Hirst et al., 2015) and adult colonic (Rodriguez‐Tapia et al., 2016) mouse enteric neurons. We did not observe morphological changes of the ENS due to tone release of the surrounding smooth musculature after nicardipine application when samples were pressed against the observation window, suggesting a direct action of this dihydropyridine compound on enteric nerves, rather than an indirect effect mediated by its effect on smooth muscle. This possibility has been put forth by earlier investigators (De Ponti et al., 1989; Marcoli et al., 1989): they found that nifedipine, verapamil and diltiazem inhibited the migrating motor complex (muscle+neural activity), but also led to a significant reduction of acetylcholine production (neural activity). Regardless of whether the effect of nicardipine is mediated by nerve, muscle or both, our results stress that this inhibitor, which is widely used in *ex vivo* physiological experiments, can have a profound impact on spontaneous ENS Ca^2+^ activity.

Mechanosensitive neurons detect mechanical distension and relay the bioelectric signal to excitatory or inhibitory effector neurons (Fung & Vanden Berghe, 2020). A broad population of enteric neurons respond to mechanical stress by a change in their firing behaviour and/or frequency (Mazzuoli & Schemann, 2012; Mazzuoli‐Weber & Schemann, 2015). Distension affects most of the neurons in the ENS by locally modulating their levels of intracellular Ca^2+^ (Cavin et al., 2023). Similar observations were made in primary cultures of neurons from the oesophagus (Dong et al., 2015). Candidate ion channels considered were Piezo1 (Drokhlyansky et al., 2020), TRP (Cavin et al., 2023), and L‐type channel and intracellular SOC, which we have shown to be involved in spontaneous Ca^2+^ activity. In the colon of adult mice, distension‐induced regulation of intracellular Ca^2+^ was shown to be dependent on K_Ca1.1_ channel activity (Cavin et al., 2023). We found that when the embryonic gut was pinched or inflated, the ENS responded with a conspicuous, transient increase in intracellular Ca^2+^. Strikingly, this response was resistant to GdCl_3_ (TRP, Piezo and K_Ca1.1_ inhibitor), 2‐APB, nicardipine and TTX, and was present even at very early embryonic stages (E14.5). It was, however, abolished in low‐calcium high‐magnesium solution that blocks synaptic transmission. We further demonstrated that this TTX‐resistant response is present in the adult colon. Our results allude to the fact that the increase of intracellular Ca^2+^ in the ENS might be a generic reaction resulting from compression‐induced disruption of the endoplasmic reticulum within cells, which triggers a Ca^2+^ release, even in the presence of 2‐APB. Similarly, electric field stimulation‐induced Ca^2+^ transients still persisted after the addition of a cocktail of antagonists to block all five types of voltage‐gated Ca^2+^ channels in E12–E18 mice (Hao et al., 2011). Our results suggest that mechanical (and perhaps electrical) stimulation can trigger Ca^2+^ release by cytoplasmic phenomena that are independent of voltage‐gated channels at the membrane. Another possibility is that of TTX‐resistant neurons, which have been reported in the ENS (Cavin et al., 2023) and CNS (Baranauskas, 2007; Bean, 2007). Although three to five times slower than the kinetics of fast TTX sensitive neurons (Baranauskas, 2007), they could provide a possible means of mechanosensation and propagation of calcium signals in the presence of TTX.

One limitation of our study is that we did not precisely control strain or strain rate, and could therefore only provide data on the presence of not of the Ca^2+^ up‐rise – further studies on the mechanisms of mechanosensation by ENS neurons should ideally address this challenge. Further, we do not know what chemical response is triggered by the Ca^2+^ up‐rise, that is, whether it is accompanied by neurotransmitter release and how it affects muscle. Finally, we have not been able to observe the neurogenic alternating high‐frequency low‐frequency contractions at E18.5 during Ca^2+^ imaging because we were pressing the sample against the Petri dish. It will be most interesting to gather these data to understand how neural activity is modulated during these cyclic events.

## AUTHOR CONTRIBUTIONS

The experimental work was performed at Laboratoire Matière & Systèmes Complexes. Richard J. Amedzrovi Agbesi performed experiments, analysed, interpreted and synthesized data, drafted and revised the paper. Amira El Merhie performed experiments and analysed data. Nick J. Spencer and Tim Hibberd analysed data, and revised the paper. Nicolas R. Chevalier supervised the project, performed experiments, interpreted data and revised the paper. All authors have read and approved the final version of this manuscript and agree to be accountable for all aspects of the work in ensuring that questions related to the accuracy or integrity of any part of the work are appropriately investigated and resolved. All persons designated as authors qualify for authorship, and all those who qualify for authorship are listed.

## CONFLICT OF INTEREST

None declared.

## Supporting information

Video S1. Ca^2+^ activity in E11.5 ileum before and after addition of TTX.

Video S2. Ca^2+^ activity in E18.5 duodenum before and after addition of veratridine and TTX.

Video S3. Alternating high‐ and low‐frequency contractions in E18.5 proximal midgut are abolished by TTX.

Video S4. Ca^2+^ activity in E18.5 duodenum before and after addition of 2‐APB.

Video S5. Ca^2+^ activity in E18.5 duodenum before and after addition of nicardipine.

Video S6. Effect of nicardipine on Ca^2+^ activity in E18.5 duodenum when the gut is free to move (not pressed against the observation window).

Video S7. Lateral view of the pinch with a force calibrated pipette

Video S8. Ca^2+^ up‐rise in the E18.5 ENS and smooth muscle contraction induced by pinching.

Video S9. Reproducibility of Ca^2+^ up‐rise in the E18.5 ENS with three consecutive pinches showing that the Ca^2+^ up‐rise is not induced by wounding.

Video S10. Resistance of the Ca^2+^ up‐rise induced by pinching in the E18.5 ENS to TTX, nicardipine, 2‐APB and GdCl_3_.

Video S11. Lumen pressurization also induces a Ca^2+^ up‐rise in the ENS of E18.5 duodenum, which is resistant to TTX.

Video S12. Pinching induced Ca^2+^ activity in the adult mouse colon (two pinches).

Video S13. Pinching induced Ca^2+^ activity in the adult mouse colon (two pinches) in the presence of TTX.

Video S14. Synaptic blocking effect in on spontaneous‐ and pinching‐induced Ca^2+^ activity in adult mouse colon.

Video S15. Inhibition of Ca^2+^ activity by nicardipine (10 μM) in adult mouse colon.

## Data Availability

All data supporting the results have been included in the manuscript and its supplementary material (videos). Additional material is available upon request.
